# *Pseudomonas syringae* DC3000 infection increases glucosylated N-glycans in *Arabidopsis thaliana*

**DOI:** 10.1007/s10719-022-10084-6

**Published:** 2022-10-21

**Authors:** Gernot Beihammer, Andrea Romero-Pérez, Daniel Maresch, Rudolf Figl, Réka Mócsai, Clemens Grünwald-Gruber, Friedrich Altmann, Els J. M. Van Damme, Richard Strasser

**Affiliations:** 1 Institute of Plant Biotechnology and Cell Biology, Department of Applied Genetics and Cell Biology, University of Natural Resources and Life Sciences, Vienna, Austria; 2 Laboratory of Biochemistry and Glycobiology, Department of Biotechnology, Ghent University, Ghent, Belgium; 3 Institute of Biochemistry, Department of Chemistry, University of Natural Resources and Life Sciences, Vienna, Austria; 4 Core Facility Mass Spectrometry, University of Natural Resources and Life Sciences, Vienna, Austria

**Keywords:** ER stress, Glycan, Glycosylation, Misfolded glycoproteins, Plant-pathogen interaction, Posttranslational modification

## Abstract

Studying the interaction between the hemibiotrophic bacterium *Pseudomonas syringae* pv. tomato DC3000 and *Arabidopsis thaliana* has shed light onto the various forms of mechanisms plants use to defend themselves against pathogen attack. While a lot of emphasis has been put on investigating changes in protein expression in infected plants, only little information is available on the effect infection plays on the plants N-glycan composition. To close this gap in knowledge, total N-glycans were enriched from *P. syringae* DC3000-infected and mock treated Arabidopsis seedlings and analyzed via MALDI-TOF–MS. Additionally, fluorescently labelled N-glycans were quantified via HPLC-FLD. N-glycans from infected plants were overall less processed and displayed increased amounts of oligomannosidic N-glycans. As multiple peaks for certain oligomannosidic glycoforms were detected upon separation via liquid chromatography, a porous graphitic carbon (PGC)-analysis was conducted to separate individual N-glycan isomers. Indeed, multiple different N-glycan isomers with masses of two N-acetylhexosamine residues plus 8, 9 or 10 hexoses were detected in the infected plants which were absent in the mock controls. Treatment with jack bean α-mannosidase resulted in incomplete removal of hexoses from these N-glycans, indicating the presence of glucose residues. This hints at the accumulation of misfolded glycoproteins in the infected plants, likely because of endoplasmic reticulum (ER) stress. In addition, poly-hexose structures susceptible to α-amylase treatment were found in the DC3000-infected plants, indicating alterations in starch metabolism due to the infection process.

**Supplementary Information:**

The online version contains supplementary material available at 10.1007/s10719-022-10084-6.

## Introduction

As sessile organisms, plants are constantly challenged by various abiotic and biotic stresses which they must cope with to ensure their survival. They have evolved strategies to fight off a wide variety of different biological pests (reviewed in [1]). As a first line of defense, plants detect certain conserved molecular structures such as bacterial flagellin via pattern recognition receptors (PRRs). These so-called microbial- or pathogen-associated molecular patterns (PAMPs or MAMPs) trigger PAMP-triggered immunity (PTI) within the plant [2]. To protect from this, some bacteria have evolved strategies to mask recognition of their flagella [3] and/or produce effectors that enhance their virulence [4]. Plants, on the other hand, have developed sophisticated methods for detection of these effector molecules [5]. Upon recognition of effectors, plants launch effector triggered immunity (ETI), which ultimately leads to programmed cell death. *Pseudomonas syringae* pv tomato DC3000 has proven an indispensable model organism for the study of this arms race between plants and bacteria [6].

In recent studies, a beneficial effect on plant resilience to *P. syringae* DC3000 infection was detected upon overexpression of a carbohydrate binding protein (lectin) from *Arabidopsis thaliana*, F-box-Nictaba [7, 8]. Plant lectins are frequently so-called chimerolectins, meaning that the protein consists of multiple domains. The lectin domain of the protein is responsible for carbohydrate binding activity while the other domain(s) can exert a different function [9]. This introduces a broad potential functional spectrum for lectin containing proteins in plants [10]. So far, the function, binding targets, and interactors of only a few of these lectins have been deciphered. The interaction between glycans and plant lectins might thus have important roles in biotic stress responses, not only by recognition of bacterial glycans but also for downstream signalling.

N-glycosylation is a conserved protein modification, whereby a preformed oligosaccharide precursor consisting of two N-acetylglucosamines (GlcNAcs), nine mannoses and three glucoses is attached to certain asparagine residues on newly formed proteins in the endoplasmic reticulum (ER). This transfer is catalyzed by the enzyme oligosaccharyltransferase (OST), a multimeric protein complex in most eukaryotes [11]. The attached oligosaccharide is subsequently modified, first by cleavage of glucoses and mannoses, and later by attachment of new sugar residues giving rise to a wide variety of species dependent N-glycan structures [12]. The N-glycan attachment is crucial for correct folding of many glycoproteins [13], can affect their stability [14] and mediate inter- and intracellular interactions [15, 16]. The role of N-glycans in human disease has been the subject of extensive research [17, 18].

In plants, various studies have reported increased susceptibility to biotic stress of mutant lines defective in various steps of N-glycosylation. An *A. thaliana* mutant line defective in Asparagine Linked Glycosylation 3 (ALG3), an enzyme required for synthesis of the N-glycan precursor, was more susceptible to *P. syringae* DC3000 infection [19]. In other studies, mutant lines of *A. thaliana* containing T-DNA insertions in Staurosporin and Temperature Sensitive 3A (STT3A), the catalytic subunit of the OST complex, and N-Acetylglucosaminyltransferase I (MGAT1/GNTI), a glycosyltransferase required for biosynthesis of complex N-glycans, were found to be more susceptible to biotic challenge [20, 21].

This study aimed at shedding light on the role of N-glycans in the response to *P. syringae* DC3000 infection in *A. thaliana*. To this end, the changes in the total N-glycan composition of infected and mock treated Arabidopsis wild type seedlings were investigated. An increased fraction of glucose-bearing as well as oligomannosidic N-glycans was detected, going hand in hand with a reduced fraction of complex or highly processed N-glycans. In addition, structures consisting of poly-glucoses were found, which are likely the result of altered starch metabolism due to the infection.

## Materials and methods

### Plant material and growth conditions

*A. thaliana* Col-0 plants were grown on half-strength MS medium supplemented with 3% (w/v) sucrose. 25 seeds were sown per plate, stratified for 48 h in the dark at 4 °C and the plants subsequently grown at 21 °C under long day conditions (16 h light/8 h dark photoperiod). After 14 days, the plants were subjected to bacterial infection via flood inoculation.

### Bacterial infection of plants

A slightly modified version of the flood inoculation protocol published by Ishiga *et al.* [22] was used for infection assays. Bacterial cultures of *P. syringae* pv tomato DC3000 were grown in King’s B medium for 24 h at 28 °C and 200 rpm agitation in baffled flasks. On the day of the infection, the OD600 of the culture was set to 0.4 and bacteria allowed to regrow until the OD600 was at 0.8. Bacteria were then centrifuged at 800 g for 5 min and resuspended in 10 mM MgSO4. The OD600 of the bacterial solution was adjusted to 0.015 and 0.025% (v/v) Silwet-77 was added. For the infection, either mock solution (10 mM MgSO4 with 0.025% Silwet-77) or bacterial inoculation solution was poured into the plates until all plants were completely covered. After 10 min of incubation, the solution was poured off, plates sealed and transferred back to the growth chamber.

### Glycan analysis in infected and mock treated plants via MALDI-TOF–MS

Total N-glycans present within infected and mock treated plants were analyzed as described previously [23]. In short, 500 mg of plant material were grinded and resuspended in 5% (v/v) formic acid solution containing 0.1 mg/mL pepsin. After incubation for 16 h at 37 °C, solids were removed via centrifugation. Enrichment of glycoproteins from the supernatant was done by cation exchange chromatography followed by a size exclusion step. Subsequently, N-glycans were cleaved off from the peptide backbone by incubating with peptide N-glycosidase A (PNGase A) for 16 h at 37 °C. On the next day, samples were acidified by adding 30% (v/v) acetic acid and applied to a cation exchange resin. The flow-through containing N-glycans was collected, concentrated, and applied to a C18 column. After elution with 1% (v/v) acetic acid, samples were concentrated under mild vacuum and reconstituted in water. Spectra were acquired on an Autoflex Speed mass spectrometer (Bruker) using 2,5-dihydroxybenzoic acid as matrix.

### Chromatographic methods and HPLC-FLD analysis

Purified N-glycans were labelled with procainamide (Supelco) according to the manufacturer’s instructions. Labelled N-glycans were resuspended in 10 µL solvent A (80 mM formic acid buffered to pH 4.4 with ammonia), and the injection volume was 1.5 µL. Separation of glycans was performed with an Agilent AdvanceBio Glycan Mapping column (2.1 × 150 mm, 1.8 μm) with a Security Guard Ultra precolumn (Phenomenex) on a Nexera X2 HPLC system with a RF-20Axs fluorescence detector equipped with a semimicro flow cell (Shimadzu, Korneuburg, Austria). Fluorescence was measured with wavelengths Ex/Em 310 nm and 370 nm. The applied gradient started with an initial hold of solvent B (85% acetonitrile in solvent A) at 99% for 8 min and a decrease to 57% solvent B over 60 min, followed by 25% solvent B in 2 min at a flow rate of 0.4 ml/min. The PGC analysis of purified N-glycan isomers was done as described previously [24].

### β-N-acetylhexosaminidase treatment

For cleavage of terminal N-acetylglucosamines, β-N-acetylhexosaminidase (Sigma-Aldrich) was used. For the reaction, purified N-glycans were resuspended in 0.1 M citrate buffer and incubated with the enzyme for 16 h at 37 °C. 

### GC–MS analysis

For analysis of poly-hexoses, infected seedlings were crushed in the presence of 5% formic acid and solids removed via centrifugation. Supernatant was loaded on a PGC cartridge and three different acetonitrile concentrations (10%, 20%, 35%) used in succession to elute different compounds. The poly-hexoses present in the 20% acetonitrile fraction were hydrolysed and subsequently peracetylated. Separation of monosaccharides was done using an Agilent J&W HP-5 ms GC Column (30 m × 0.25 mm, 0.25 μm) installed in a GC–MS system (GC 7820 A & MSD 5975, Agilent).

### α-amylase treatment

Digest of poly-glucoses via α-amylase from *Aspergillus oryzae* (Sigma-Aldrich) was conducted according to manufacturer’s instructions. For the mock treated control, the α-amylase was inactivated by heating to 95 °C for 15 min. After incubation, digested and mock treated samples were purified using a PGC-cartridge.

## Results

### *P. syringae* DC3000 infection alters the plants overall N-glycan pattern

Changes in the N-glycan profile of *A. thaliana* Col-0 plants were analyzed upon infection with *P. syringae* DC3000. 14-day old Col-0 wild-type seedlings were infected using a flood-inoculation assay. The infected seedlings displayed first symptoms of infection at 3 days post infection (dpi) such as damaged leaves and chlorosis, which were even more pronounced at 5 dpi (Fig. S1). Mock-treated plants on the other hand showed no disease symptoms.

**Fig. 1:**
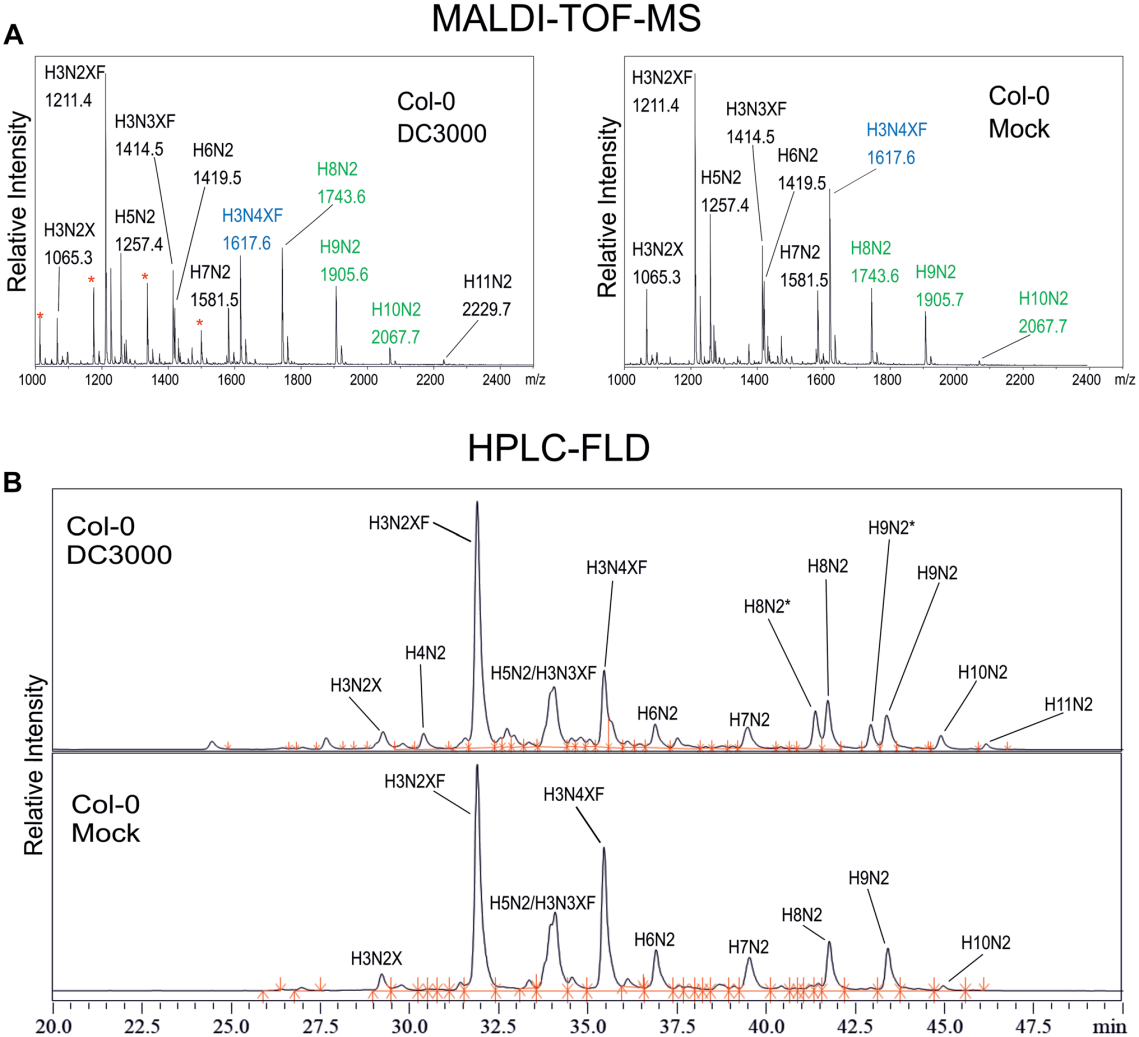
Mass spectrometric analysis of total N-glycans and chromatographic separation of fluorescently labeled N-glycans present in seedling leaves of mock-treated and DC3000-infected Col-0 wild type plants. **A** MALDI-TOF–MS analysis of purified N-glycans. Samples were harvested at 3 dpi, total N-glycans purified and analyzed via MALDI-TOF–MS in positive deflector mode. Peaks appear mainly as sodium adducts. Red asterisks indicate peaks with masses corresponding to poly-hexoses. H3N4XF is highlighted in blue. H8N2, H9N2 and H10N2, respectively, are highlighted in green. A representative spectrum of three independent biological replicates is shown. **B** HPLC-FLD analysis of total N-glycans. Purified N-glycans were labelled with procainamide and measured using HPLC with fluorescence detection (HPLC-FLD). Two peaks with masses corresponding to H8N2 and H9N2, respectively, were detected in the infected samples. The additional peaks were denominated H8N2* and H9N2*. A representative spectrum of four independent biological replicates is shown. H: Hexose, N: N-Acetylhexosamine, X: Xylose, F: Fucose

For analysis of total N-glycans, the aerial parts of the plants were harvested at 1, 3 and 5 dpi, N-glycans enriched and subsequently analyzed via MALDI-TOF–MS (Figs. 1A and S2). In accordance with the onset of disease symptoms, the differences between samples from infected and mock treated plants were more pronounced at 3 and 5 dpi. The fraction of H3N4XF glycans (highlighted in blue), most likely corresponding to the complex N-glycan structure GnGnXF (a schematic representation of N-glycan abbreviations is shown in Fig. S3), appeared to be lower in the infected plants. On the other hand, the MS-spectra from the mock treated plants displayed a reduced signal of H8N2, H9N2 and H10N2 glycans (highlighted in green), likely corresponding to oligomannosidic N-glycans. In addition, peaks with masses corresponding to H11N2 were detected in DC3000-infected samples harvested at 3 dpi, which were absent in the mock treated controls. These structures could correspond to Man9 structures still bearing 2 glucoses.

Interestingly, additional peaks were present in the samples from DC3000-infected plants (red asterisks in Figs. 1A and S2). The additional peaks had masses corresponding to poly-hexoses. While these peaks were present in all samples from DC3000-infected plants, they were absent in the mock treated controls.

**Fig. 2:**
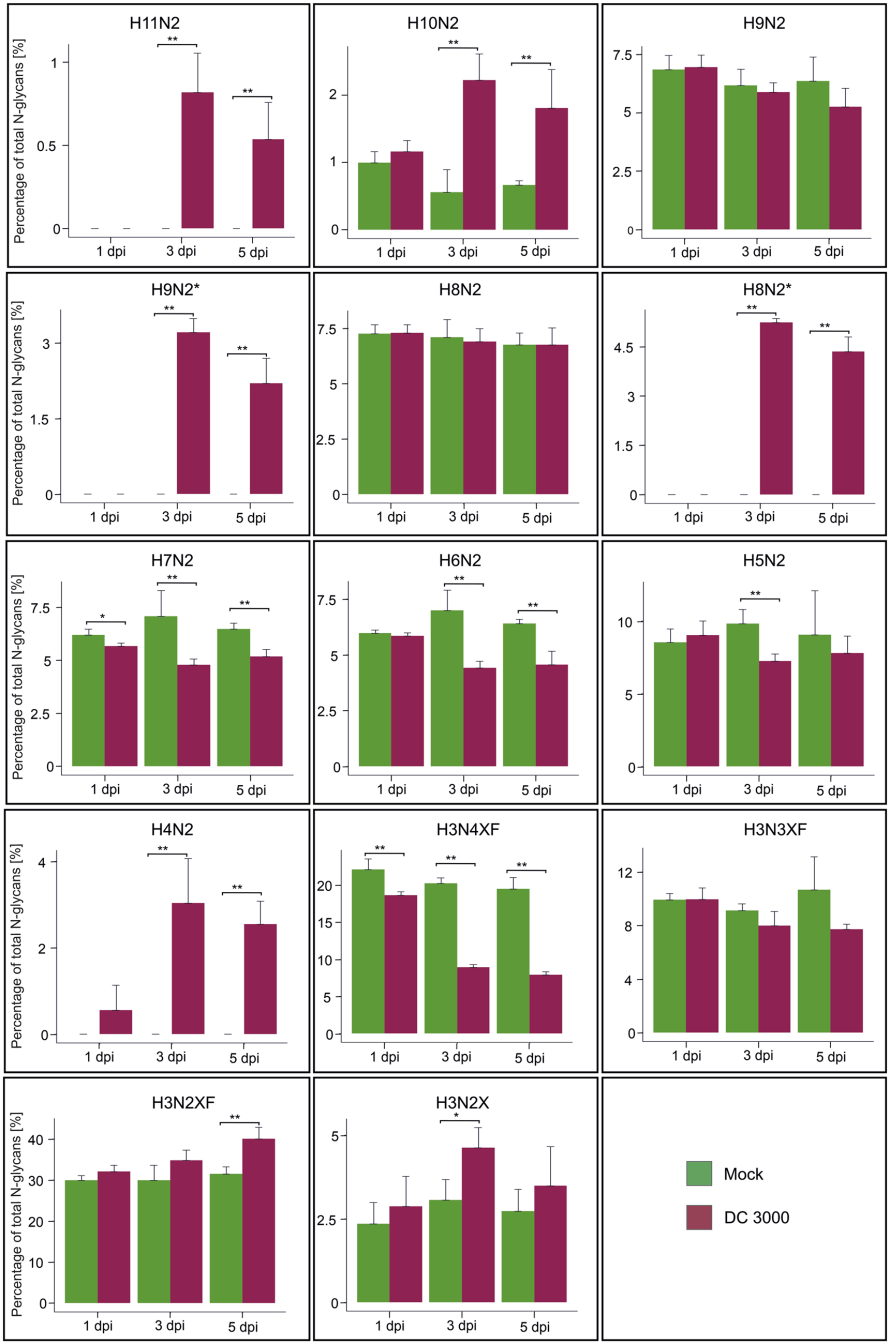
Quantification of individual N-glycans in mock-treated and DC3000-infected Col-0 plants. The Y-axis shows percentage of the individual glycoforms with respect to total detected N-glycans. The quantification of individual N-glycans is based on the HPLC-FLD measurement. Error bars indicate standard deviation of four independent biological replicates. Asterisks indicate statistically significant differences between infected and mock treated plants harvested at the same time point based on a student’s t-test (* *p* < 0.05; ** *p* < 0.01). H: Hexose, N: N-Acetylhexosamine, X: Xylose, F: Fucose

To quantify the individual glycoforms, released and purified N-glycans were labeled with procainamide and measured via HPLC-FLD (Fig. 1B). Individual peaks were identified via LC–ESI–MS analysis (Fig. S4A). As H5N2 and H3N3XF (likely Man5 and GnMXF) eluted as one peak, a β-hexosaminidase digest was conducted to allow quantification of the peaks corresponding to H5N2 and H3N3XF, respectively (Fig. S4B). Interestingly, glycans with masses corresponding to H8N2 and H9N2 eluted as two separate peaks in the samples from DC3000-infected plants, while only one peak could be observed in the mock controls. The additional earlier eluting peaks were denominated H8N2* and H9N2*, respectively, and quantified separately. Based on the results of the HPLC-FLD measurements, the amounts of the individual N-glycans were quantified in mock treated and infected Col-0 at 1, 3, and 5 dpi (Fig. 2; Table S1). Samples from infected plants contained elevated levels of H10N2 at 3 and 5 dpi as well as an increase in H3N2XF structures at 5 dpi and H3N2X structures at 3 dpi. In addition, H4N2, H8N2* and H9N2* as well as H11N2 glycans were only detected in the infected samples. In contrast to wild-type, infected samples displayed significantly reduced H6N2 levels at 3 and 5 dpi. H7N2 and H4N3XF structures were slightly reduced in infected samples harvested at 1 dpi but showed severe reduction at 3 and 5 dpi. No differences between mock treated and infected plants could be observed for H9N2, H8N2 and H3N3XF structures.

Changes in the relative amounts of individual N-glycans over time were more pronounced in the infected plants compared to mock treated plants (Fig. S5). While in mock treated plants only the amounts of H10N2 were altered, significant changes could be observed for all detected N-glycans except for H8N2 in the infected plants. For most structures, the differences were more pronounced between 1 and 3 dpi compared to the differences between 3 and 5 dpi.

### *P. syringae* DC3000-infected plants contain N-glycans not present in mock treated plants

Based on the results from the MALDI-TOF–MS analysis of total N-glycans, there appeared to be an increase in H8N2 and H9N2 structures in infected samples, but upon HPLC-separation it became apparent that the peaks in the MALDI-spectra are derived from multiple glycoforms with equivalent masses. Thus, to further investigate the nature of the H8N2* and H9N2* peaks, purified N-glycans from mock treated and infected Col-0 plants were separated on a porous graphitic carbon (PGC) column followed by LC–ESI–MS analysis. The use of a PGC column allows separation of different N-glycan isomers [24]. An external N-glycan standard from *Canavalia ensiformis* (jack bean) was included as a reference for identification of peaks. In accordance with the HPLC-FLD measurement, in which H5N2, H6N2 and H7N2 eluted as one peak in both control and infected samples, no differences were detected for these glycoforms (Figs. S6 and S7**)**. H7N2 structures showed only poor separation, but three peaks could be identified in samples from both mock-treated and DC3000-infected seedlings eluting at similar retention times as the glycans in the reference sample.

**Fig. 3:**
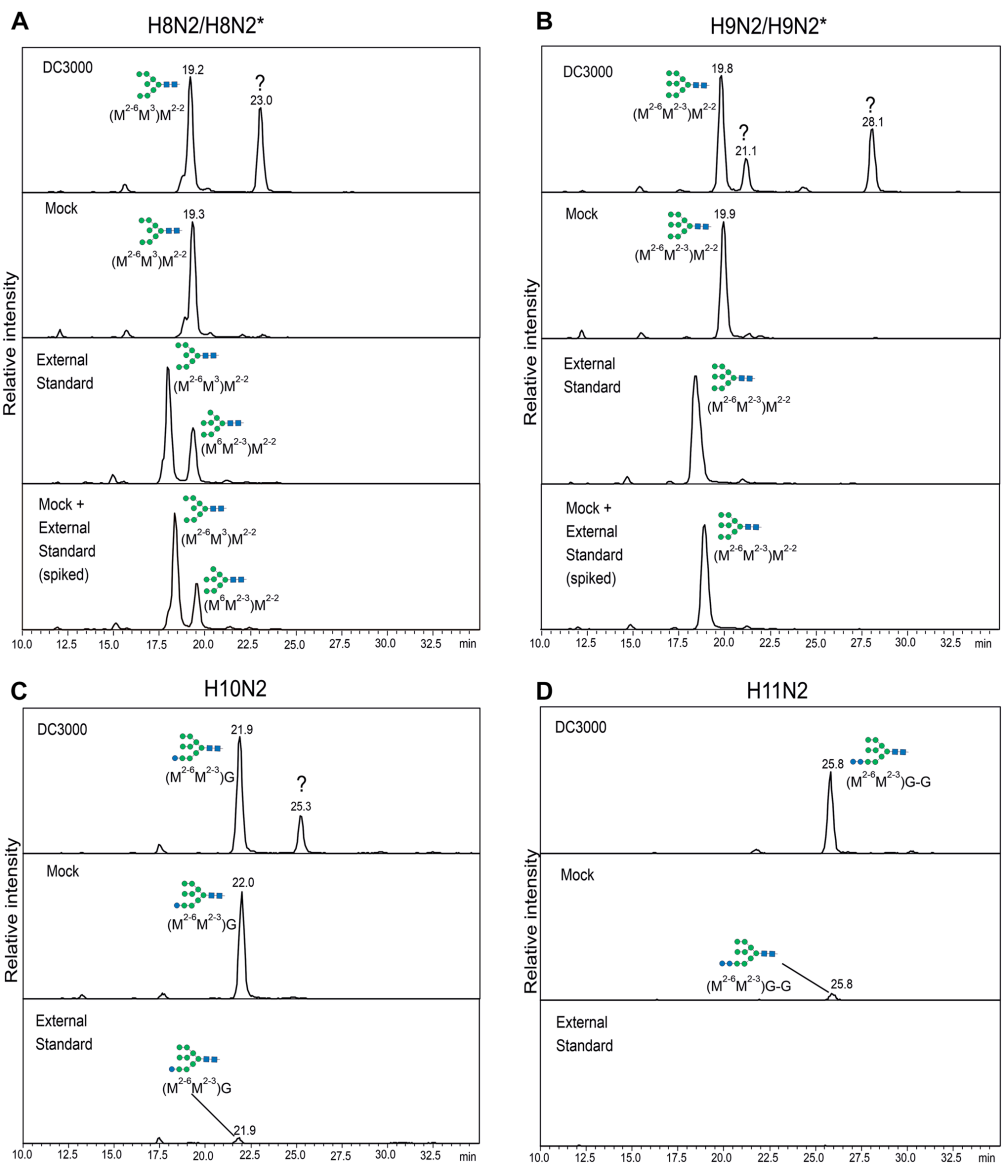
PGC-LC–ESI–MS analysis of N-glycan isomers present in mock treated and DC3000-infected samples. Samples were harvested from Col-0 wild-type plants at 3 dpi, N-glycans were purified and separated via a PGC-column. An N-glycan standard from *C. ensiformis* was included as reference. The N-glycan nomenclature is according to the Proglycan system (www.proglycan.com). H: Hexose, N: N-Acetylhexosamine, X: Xylose, F: Fucose. Symbols are used according to the suggestions from the Consortium for Functional Glycomics (http://www.functionalglycomics.org/)

Comparison of H8N2 glycans showed an additional peak eluting after 23.0 min in the infected sample, which was absent in the mock control (Fig. 3A) and the external standard which contains two H8N2 isomers. Overall, the elution times in the external standard were slightly shifted, most likely due to matrix effects. Spiking the mock control with external standard led to an increase of the earlier eluting peak, indicating that this was the major peak in the mock sample. The second peak in the DC3000-infected sample eluted clearly later than the second peak in the external standard, which corresponds to a H8N2 isomer not found in leaves from *A. thaliana* seedlings. The peak eluting after 23.0 min in the DC3000 sample is thus most likely a glycoform absent in both external standard and mock control.

A similar pattern was observed for H9N2 (Fig. 3B), for which the external standard contains one isomer corresponding to the peak present in the mock control. Two additional peaks were observed in the sample from infected plants eluting at 21.1 and 28.1 min, indicating the presence of three different glycoforms with masses of H9N2. Furthermore, at the mass corresponding to H10N2, an additional second peak eluting at 25.3 min could be detected in the infected sample which was absent in the mock control (Fig. 3C). The external standard contains only a minor fraction of H10N2 and is devoid of H11N2, for which the peak was a lot more pronounced in the infected sample compared to the mock control (Fig. 3D).

The presence of additional N-glycan-isomers with masses of 2 HexNAcs + 8, 9 and 10 hexoses, respectively, could be due to an increased abundance of N-glycans still bearing glucose-residues. The additional H8N2* peak might thus actually correspond to Man7 + 1 Glc or Man6 + 2 Glc, while the two additional H9N2* peaks correspond to Man8 + 1 Glc and Man7 + 2 Glc.

**Fig. 4:**
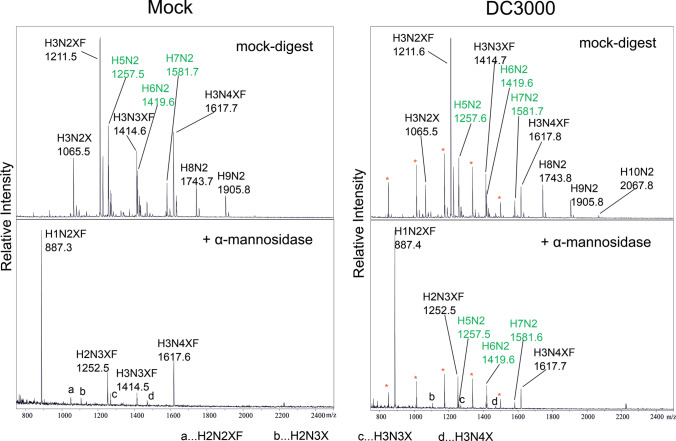
MALDI-TOF–MS analysis of N-glycans purified from mock treated and DC3000-infected Col-0 wild-type plants digested with jack bean α-mannosidase. Samples were harvested at 3 dpi, N-glycans purified, subjected to α-mannosidase digestion and measured via MALDI-TOF–MS. Red asterisks indicate peaks with masses corresponding to poly-hexoses. H5N2, H6N2 and H7N2 are highlighted in green. H: Hexose, N: N-Acetylhexosamine (HexNAc), F: Fucose, X: Xylose

To test whether the H8N2* and H9N2* peaks in the infected plants were caused by the presence of glucose-bearing N-glycans, the purified N-glycans were digested with jack bean α-mannosidase (Fig. 4). A clear difference could be seen between samples from mock treated and infected plants: while samples from mock treated plants contained a glycan with the mass of 1 hexose + 2 N-acetylhexosamine + 1 deoxyhexose + 1 pentose (H1N2XF, most likely UUXF, Fig. S3) as dominant glycoform and only minor fractions of other N-glycans (H3N3XF and H2N2XF, most likely GnMXF and UMXF), peaks with masses of H5N2, H6N2 and H7N2 (highlighted in green) were detected in the DC3000-infected samples. This is in agreement with previous reports indicating that glucose bearing N-glycans are only partially susceptible to α-mannosidase digestion [25, 26].

### The poly-hexoses in the infected samples consist of glucose and are cleaved by α-amylase

Besides the presence of additional N-glycan structures, the other intriguing observation was the presence of poly-hexoses in the infected samples. These structures were detected in all infected samples harvested at 3 and 5 dpi. As the fraction of purified N-glycans used for MALDI-TOF–MS analysis should only contain N-glycans cleavable by peptidyl N-glycosidase A (PNGase A), these poly-hexoses were likely present in high concentration in the original samples and were carried over to the fraction of enriched N-glycans.

**Fig. 5:**
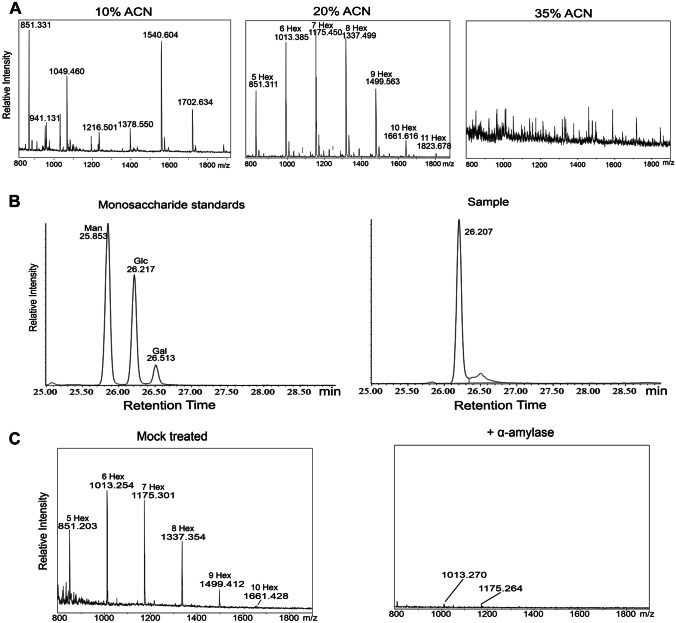
**A** Enrichment of poly-hexoses using a PGC-column. Seedlings from Col-0 wild type infected with DC3000 were harvested at 3 dpi, crushed, extracted with 5% formic acid and loaded on a PGC-cartridge. Different concentrations of acetonitrile were employed to elute the poly-hexoses. **B** GC–MS analysis of poly-hexoses present in the infected samples. Poly-hexoses from the 20% acetonitrile fraction were hydrolyzed, peracetylated and retention time compared to various monosaccharide standards. **C** MALDI-TOF–MS analysis of poly-hexoses digested with α-amylase. Enriched poly-hexoses from the 20% acetonitrile fraction were subjected to an α-amylase digest

To test whether the poly-hexoses could be purified from the original sample, seedling leaves were homogenized, resuspended in 5% formic acid, centrifuged and the supernatant loaded to a PGC-column. Elution at different acetonitrile concentrations resulted in a fraction that eluted with 20% acetonitrile containing mainly poly-hexoses (Fig. 5A). The poly-hexoses in the 20% acetonitrile fraction were then hydrolyzed and measured via GC–MS. The monosaccharides eluted as one distinct peak, indicating that only one type of monosaccharide was present in the sample. Comparison to monosaccharide standards showed that the retention time of the peak in the sample matched that of glucose (Fig. 5B). An α-amylase digest was conducted, leading to the near absence of detectable poly-glucose structures (Fig. 5C), indicating that the poly-glucoses in the infected plants stem from differences in starch metabolism.

## Discussion

The aim of this study was to monitor changes in the N-glycan profile of *A. thaliana* seedlings upon infection with *P. syringae* DC 3000. Several reports have been published focusing on changes in transcript and protein levels upon biotic challenge [27–29] or salicylic acid treatment [30, 31] in plants but so far, limited information is available with respect to changes on N-glycans [32]. Various proteins involved in pathogen recognition and other defense responses are heavily glycosylated and require the presence of N-glycans to properly exert their functions [20]. Moreover, plants have numerous lectins that play a role during biotic stress and some of them could interact with specific types of N-glycans present on plant glycoproteins [7–9]. Shedding light on the changes of the N-glycome might thus provide valuable information to develop strategies to produce disease resistant crops and to obtain an overall deeper understanding of the interplay between plants and pathogens.

Here, we focused on changes in the total N-glycan profile and did not consider potential changes in protein expression or changes in N-glycosylation efficiency on specific glycoproteins. Part of the observed differences in N-glycan composition is likely due to an altered expression pattern of plant glycoproteins in response to *P. syringae* DC3000 infection. Overall, N-glycans on plant glycoproteins appeared to be less processed in infected plants compared to mock controls. N-glycans with masses of 2 HexNAcs + 11 hexoses (H11N2, most likely Man9 structures still bearing two glucoses) were detected exclusively in the infected plants. Furthermore, structures with masses of 2 HexNAcs + 10 hexoses (H10N2) were overrepresented in the DC3000-infected plants. Separation of N-glycan isomers via a PGC column resulted in the detection of two peaks for this mass, of which one corresponds to Man9 structures decorated with one glucose. The second, later eluting peak could be Man8 + 2 glucoses, a structure that was not detected in the mock plants and is absent in the reference sample from *C. ensiformis*. LC–ESI–MS analysis yielded two peaks for structures consisting of 2 HexNAcs + 8 or 9 hexoses (H8N2 and H9N2) in the DC3000-infected plants. PGC-MS analysis of these N-glycans indicated the presence of three (in the case of H9N2) and two (H8N2) distinct N-glycan isomers, while only one isomer of each can be found in the mock controls. An α-mannosidase digest led to incomplete cleavage of glycans in the infected samples, hinting at the presence of glucoses on these additional N-glycan isomers. An increase in the amounts of mannosidic N-glycans was also detected by Jia *et al.*, 2020 [32] who analyzed changes in N-glycan composition upon syringe infiltration of leaves from 30-day-old *A. thaliana* with *P. syringae* DC 3000.

The detection of glucosylated structures suggests the accumulation of misfolded glycoproteins. Under standard conditions, glucose residues are cleaved from the N-glycans of newly formed glycoproteins during folding. This process constitutes part of a conserved quality control mechanism. In case a protein is misfolded, glucoses are reattached by the enzyme UDP-glucose:glycoprotein glucosyltransferase (UGGT) and subsequently bound by the chaperones calnexin and calreticulin to promote folding [33]. If a glycoprotein is terminally misfolded, it is cleared by the ER-associated degradation (ERAD) pathway [13]. Previous studies have reported an increased expression of proteins required for protein folding upon salicylic acid treatment [30]. Saijo *et al.* reported increased colonization with *P. syringae* DC3000 in Arabidopsis plants with defective calreticulin 3 (CRT3) [34]. This increased susceptibility was attributed to the inability to produce correctly folded EFR receptor. Coherently, increased susceptibility to DC3000 infection was also reported for T-DNA insertion lines of Arabidopsis calreticulin 2 (CRT2) [35].

In addition, induction of inositol-requiring enzyme1 (*IRE1*) a and b transcription has been reported upon biotic challenge and salicylic acid treatment [36]. The ER stress sensors IRE1a and IRE1b cause unconventional splicing of *bZIP60* mRNA. This in turn allows the translated bZIP60 protein to enter the nucleus, where it exerts its function as transcription factor to induce translation of proteins involved in the Unfolded Protein Response (UPR) [37]. The induction of the UPR has been shown to be required for conferring resistance to diverse biotic stresses [38, 39].

Interestingly, no change in the abundance of Man8 and Man9 (H8N2 and H9N2) was observed. On the other hand, DC3000-infected plants contained reduced levels of Man7 (H7N2) and Man6 (H6N2) and also reduced Man5 (H5N2) levels at 3 dpi. These N-glycan structures are generated by the α-mannosidases MNS1 to MNS3. A recent study focusing on the subcellular localization of the α-mannosidase MNS3 required for processing of Man9 to Man8 showed that MNS3 resides like MNS1 and MNS2 in the *cis*-Golgi in *A. thaliana* [23]. Based on these findings, increased retention of proteins in the ER could prevent trimming by MNS1 to MNS3 and contribute to the decreased amounts of Man5 to Man7 structures. Alternatively, the subcellular localization, protein abundance or enzymatic activity of the α-mannosidases could be altered upon *P. syringae* DC 3000 infection. The severely reduced content of GnGnXF (H3N4XF) is in line with the observed reduced processing of N-glycans. Previously, it had been reported that the lectin F-box-Nictaba, which upon overexpression confers decreased susceptibility of *A. thaliana* plants to DC3000 infection [7, 8], binds to N-acetyllactosamine structures on a glycan array [40]. Given the lack of structures higher elongated than GnGnXF detected in seedling leaves, it appears likely that the beneficial effect of F-box-Nictaba overexpression is due to a different, so far unknown, mechanism.

Besides the observed changes in the N-glycosylation profile, high amounts of poly-hexoses susceptible to α-amylase cleavage were detected in the infected plants. *P. syringae* DC3000 suppresses efficient deposition of callose within the infected plants [41]. It is thus likely that the observed poly-glucoses in the infected samples correspond to starch, even though a residual activity of the α-amylase employed in this study towards β-linked glucoses cannot be ruled out.

Previous studies have reported starch accumulation in plants as a result of biotic challenge [42–44]. Intriguingly, reduced growth of *P. syringae* was observed in an Arabidopsis mutant line containing increased levels of glucose due to alterations in starch homeostasis [45]. On the other hand, induction of an α-amylase encoded by At4g25000, AMY1, has been reported in the apoplast of leaves of *A. thaliana* upon biotic and abiotic stress [46]. However, the exact reason for the presence of poly-glucoses susceptible to α-amylase cleavage in the infected plants is unclear. In summary, our data show that *P. syringae* DC 3000 infection in leaves of *A. thaliana* seedlings leads to the formation of incompletely processed oligomannosidic N-glycans that are indicative of protein misfolding in the ER. Future studies will aim to identify the underlying mechanism and affected glycoproteins that could be misfolded upon infection.

## Supplementary Information

Below is the link to the electronic supplementary material.

Supplementary file1 (PDF 1240 KB)

## Data Availability

The data that support the findings of this study are available from the corresponding author upon reasonable request. The raw mass spectrometry files (MALDI-spectra of infected and mock treated samples harvested at 1, 3, and 5 dpi) for this study have been made publicly available at GlycoPOST (https://glycopost.glycosmos.org/, accession ID GPST000288, (https://glycopost.glycosmos.org/preview/519801245632db71b15653; PIN: 1680) [47] and have been reported according to the MIRAGE guidelines [48, 49].
