# Supplementary material for: Pseudomonas syringae DC3000 infection increases glucosylated N-glycans in Arabidopsis thaliana
Source: Glycoconj J. 2022 Oct 21;40(1):97–108. doi: 10.1007/s10719-022-10084-6 (PMC9925501; doi:10.1007/s10719-022-10084-6)
Supplement: Supplementary file 1 — Supplementary file1 (PDF 1240 KB) [file 10719_2022_10084_MOESM1_ESM.pdf]

Supplemental information for

Pseudomonas syringae* DC3000 infection increases glucosylated N-glycans in *Arabidopsis thaliana

Contents

Figure S1. Images of infected and mock treated Col-0 wild-type plants taken 1, 3 and 5 dpi.

Figure S2. MALDI-TOF-MS analysis of total N-glycans in infected and mock treated Col 0 plants 1 and 5 dpi.

Figure S3. Schematic representation of N-glycan abbreviations used in this study.

Figure S4. MS-analysis of procainamide labeled N-glycans from Col-0 seedlings either mock treated or DC3000-infected.

Figure S5. Changes in the relative abundance of individual N-glycans over time in mock-treated and DC3000-infected plants.

Figure S6. PGC-analysis of H5N2 and H6N2 glycans from Col-0 wild-type seedlings either mock-treated or DC3000-infected**.**

Figure S7. PGC-analysis of H7N2 glycans from Col-0 wild-type seedlings either mock-treated or DC3000-infected.

Table S1. List of relative amounts of individual N-glycans in infected and mock treated Col-0 wild-type plants at 1, 3 and 5 dpi.

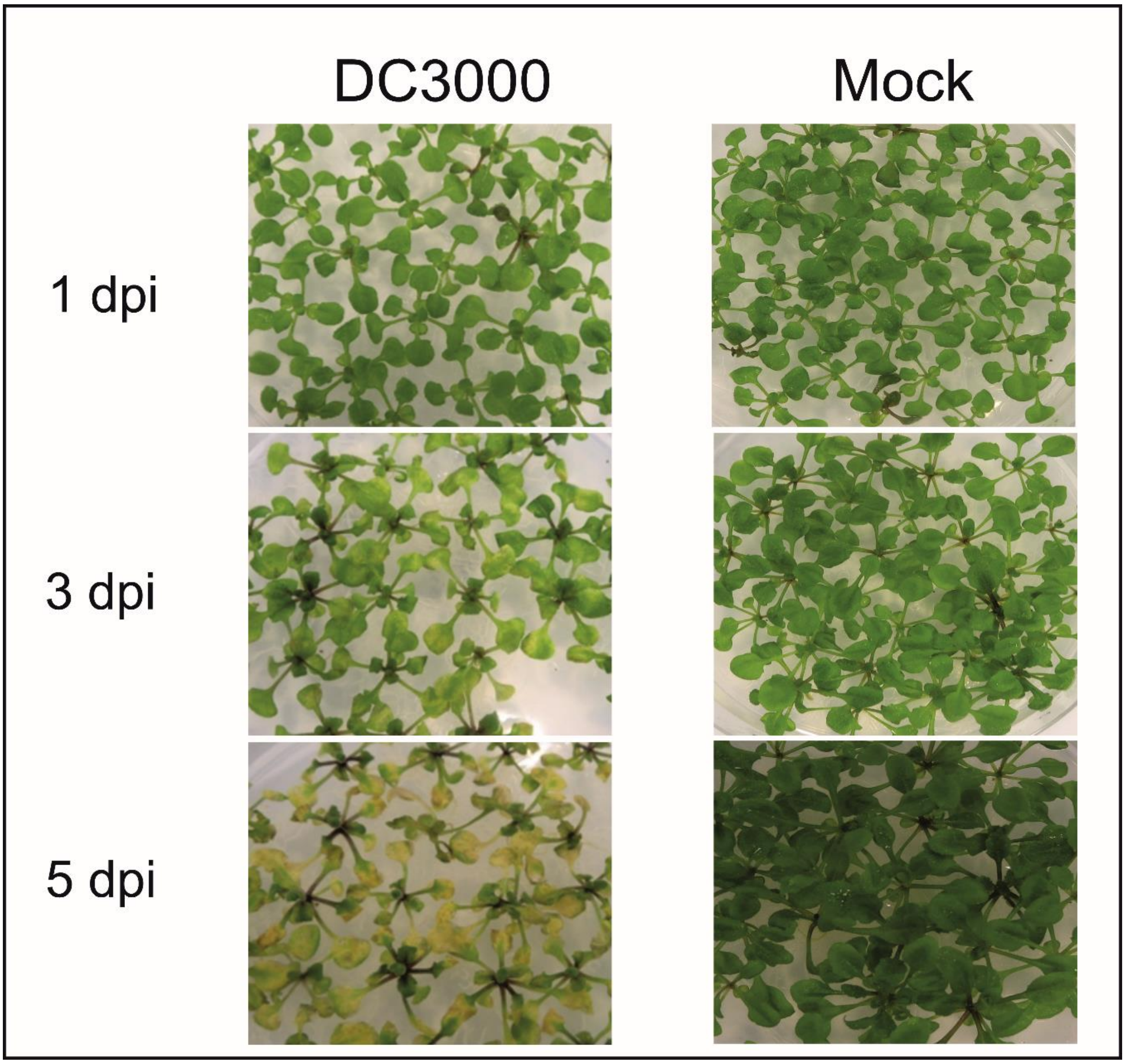

Figure S1. Images of infected and mock treated Col-0 wild-type plants taken 1, 3 and 5 dpi. Arabidopsis Col-0 seeds were sterilized, stratified for three days and grown on half-strength MS-medium for 14 days. One part of the plants was infected with *P. syringae* DC3000 using the flood inoculation assay, while the other part was mock treated with $MgSO_4$ solution.

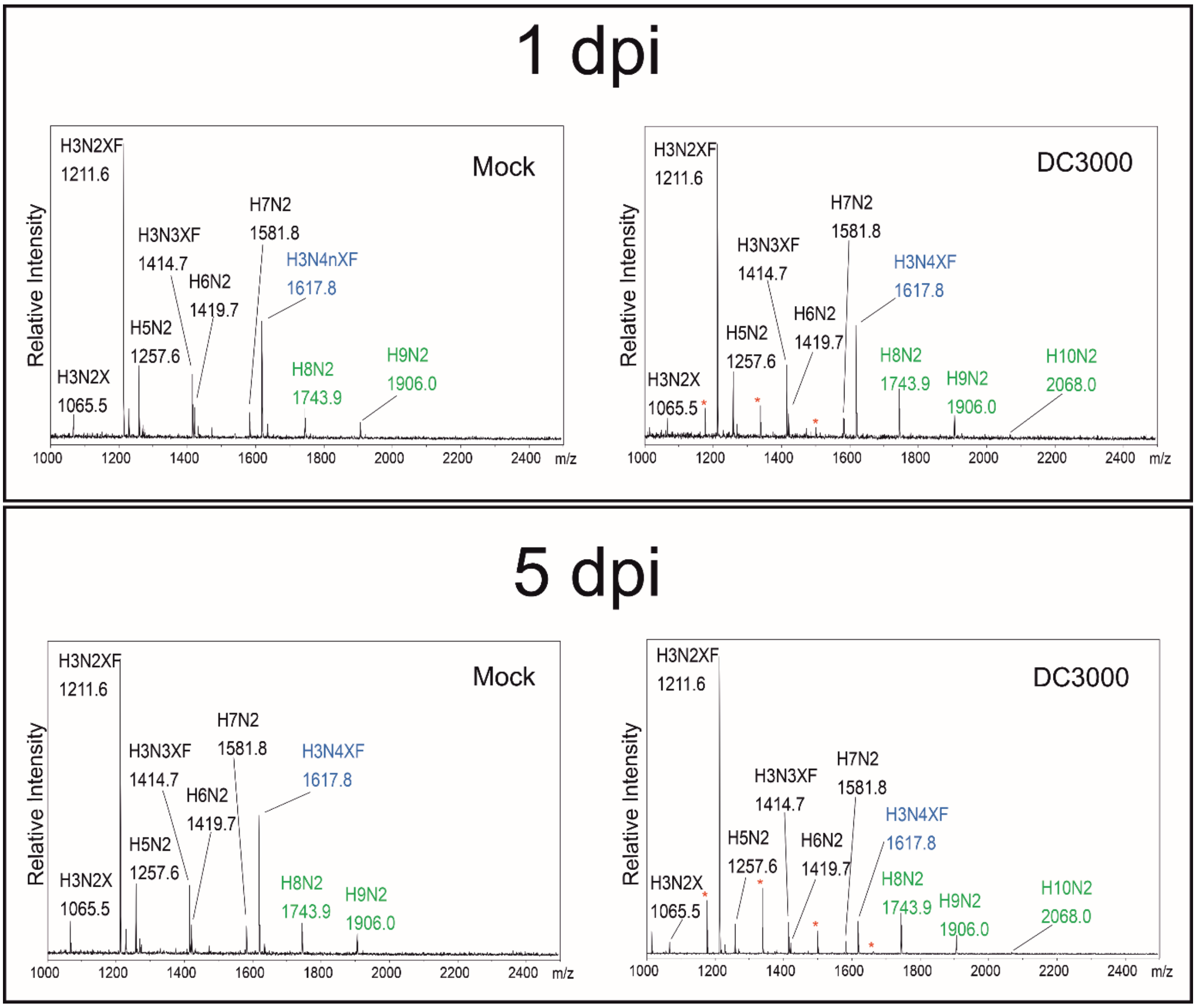

Figure S2. Representative MALDI-TOF-MS spectra of total N-glycans in infected and mock treated Col-0 plants harvested 1 and 5 dpi, respectively. 2-week-old Arabidopsis seedlings were either DC3000-infected or mock treated. Samples were frozen, grinded and N-glycans enriched. Red asterisks indicate peaks with masses corresponding to poly-hexoses. H3N4XF is highlighted in blue. H8N2, H9N2A and H10N2, respectively, are highlighted in green. H: Hexose, N: N-Acetylhexosamine, X: Xylose, F: Fucose.

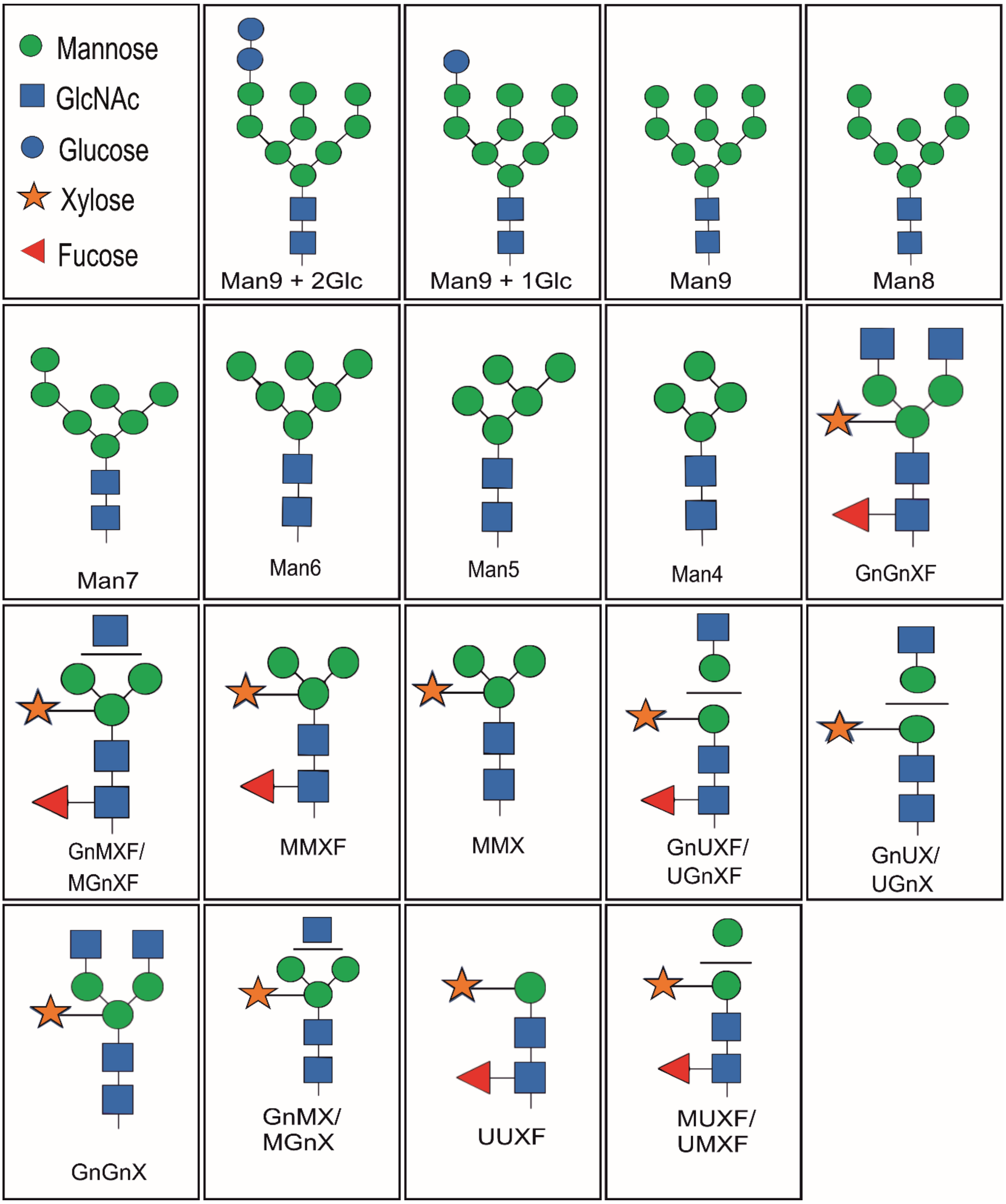

Figure S3. Schematic representation of N-glycan structures and abbreviations used in this study. The nomenclature is according to the Proglycan system (www.proglycan.com).

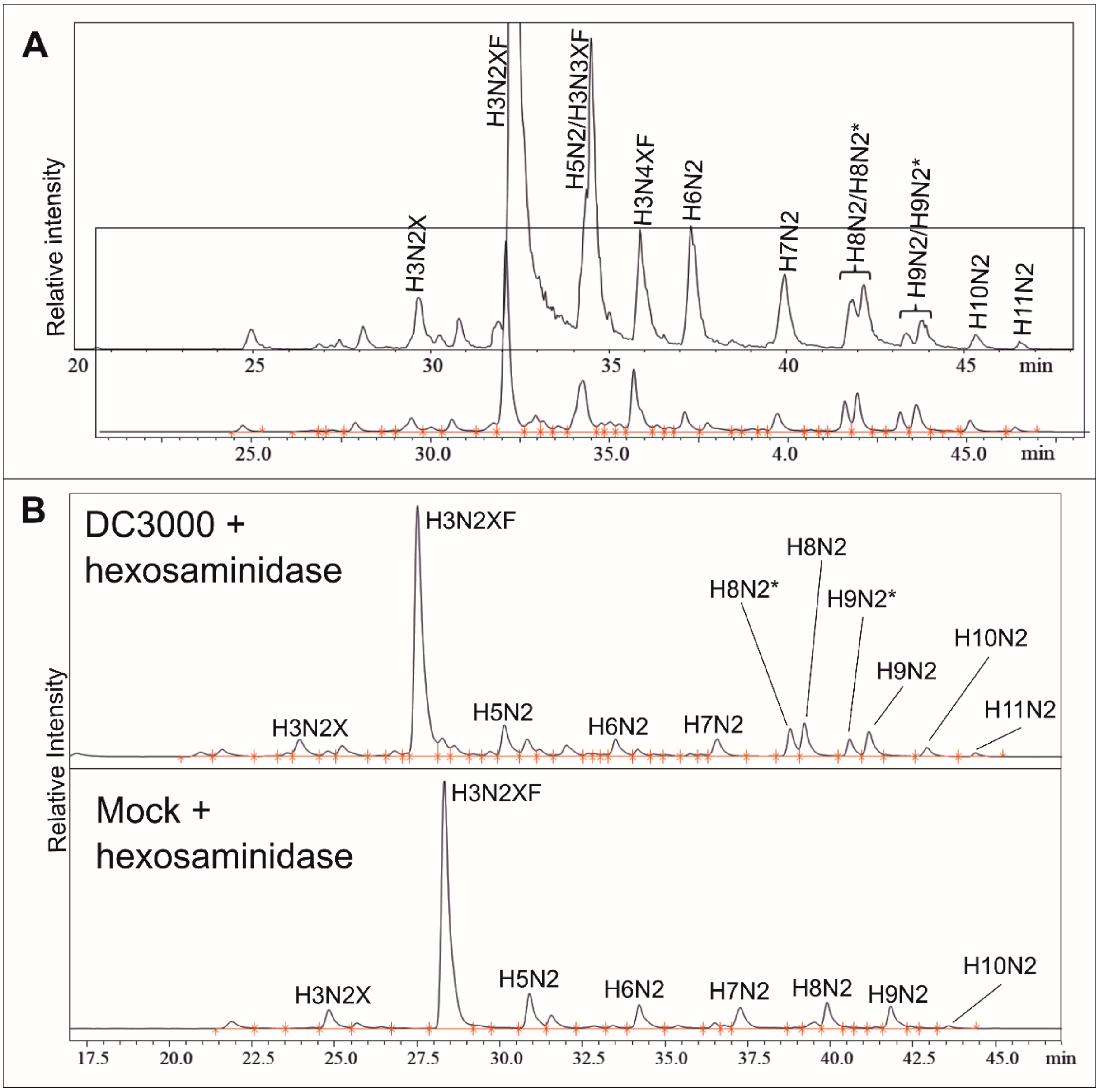

Figure S4. Analysis of procainamide labeled N-glycans from Col-0 seedlings either mock treated or DC3000 infected. (A) LC-ESI-MS analysis of procainamide labelled N-glycans from Col-0 wild type seedlings infected with *P. syringae* DC3000 harvested 3 dpi to identify which peak corresponds to which N-glycan. The lower spectrum represents the results of HPLC-FLD analysis while the upper spectrum shows the peaks obtained from LC-ESI-MS measurement. Retention times of the two spectra are aligned. (B) HPLC-FLD spectrum of either mock-treated or DC3000-infected Col-0 seedlings harvested 3 dpi upon digestion with β-N-acetylglucosaminidase (hexosaminidase) to allow quantification of H5N2 and H3N3XF glycans. H: Hexose, N: N-Acetylhexosamine, X: Xylose, F: Fucose.

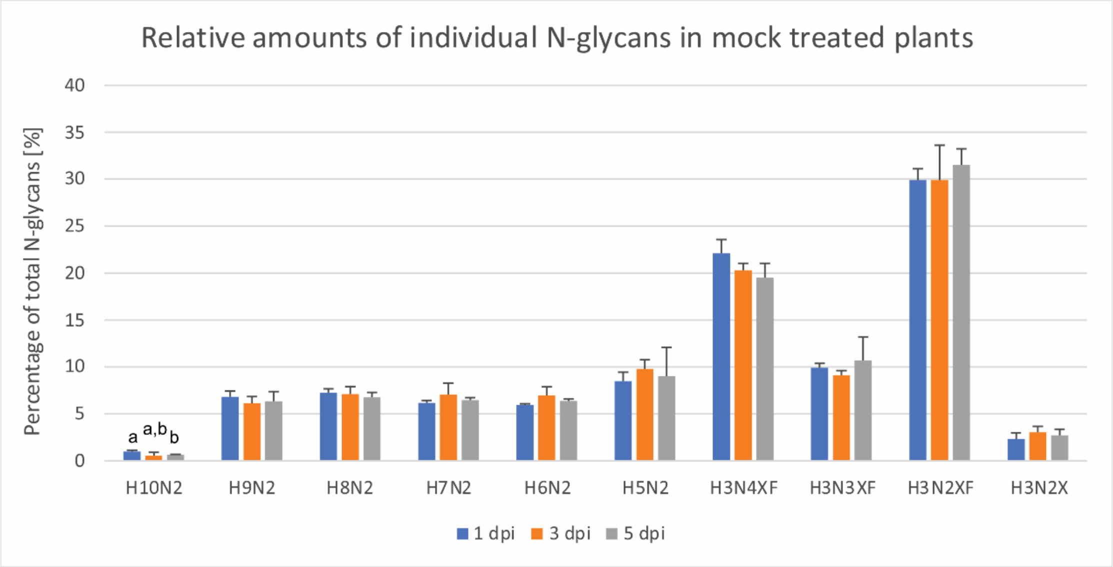

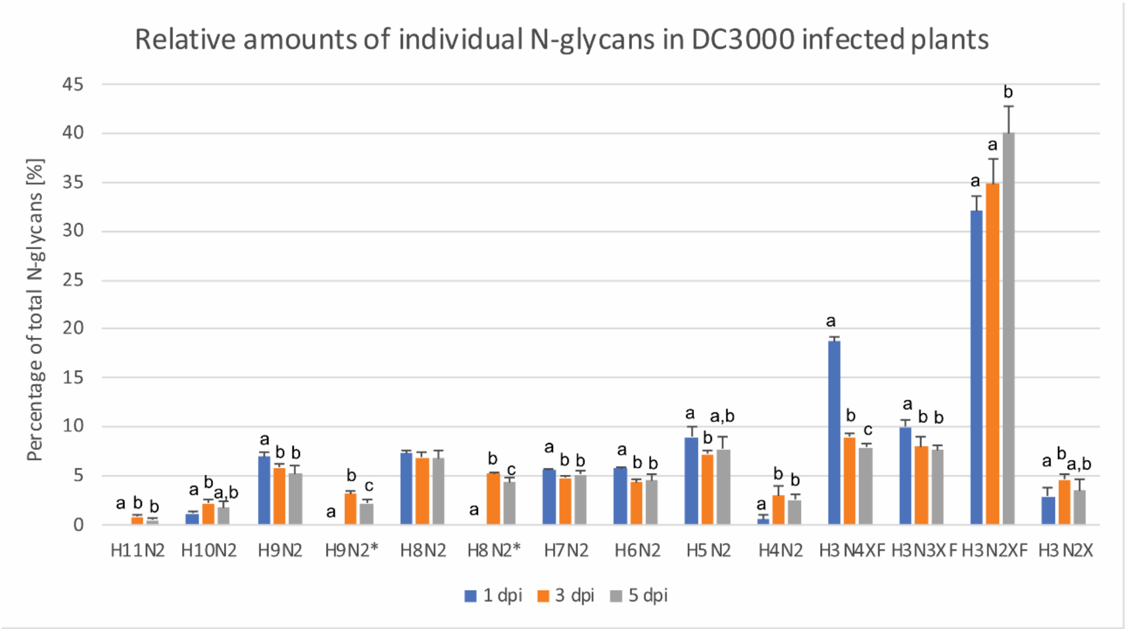

Figure S5. Changes in the relative abundance of individual N-glycans over time in mock-treated and DC3000-infected plants. Total N-glycans were purified from mock treated and infected plants harvested at 1,3 and 5 dpi, respectively, and fluorescently labelled. The quantification is based on the HPLC-FLD measurement. Error bars indicate standard deviation of four independent biological replicates. Letters (a,b,c) indicate statistically significant differences between relative amounts of the respective N-glycan at different time points based on the results of a student's t-test ($p < 0.05$).

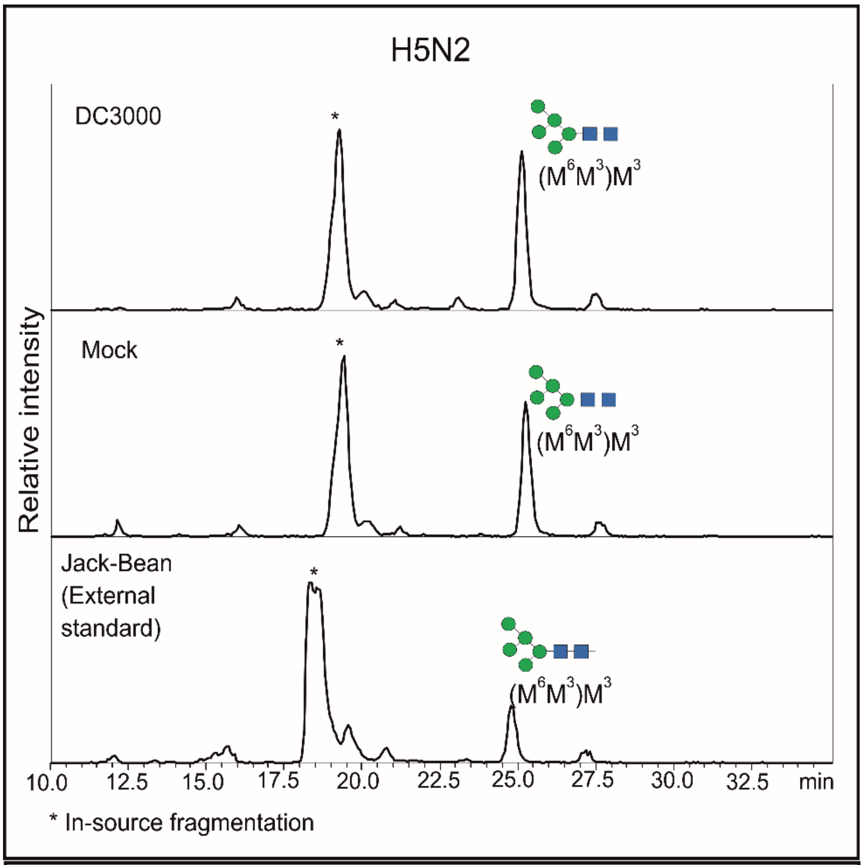

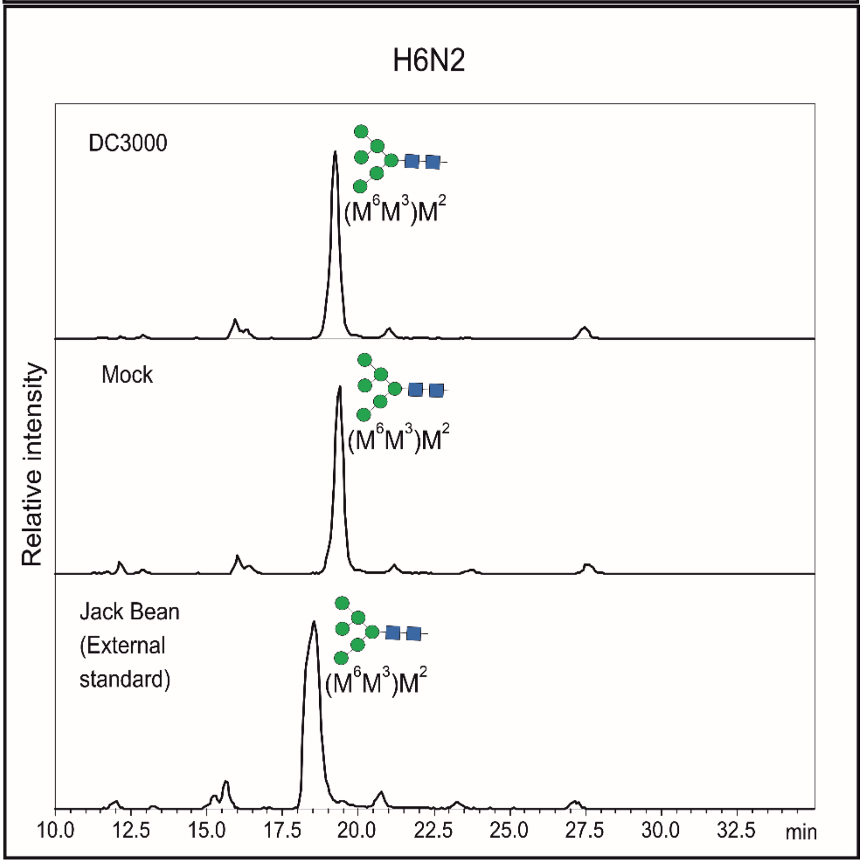

Figure S6. PGC-MS analysis of H5N2 and H6N2 glycans from Col-0 wild-type seedlings either mock-treated or DC3000-infected. The slight shift in the analyzed standards is likely derived from matrix effects. Samples were harvested 3 dpi and N-glycans purified. N-glycans from jack bean were used as external standards. N-glycan nomenclature is according to the Proglycan system (www.proglycan.com).

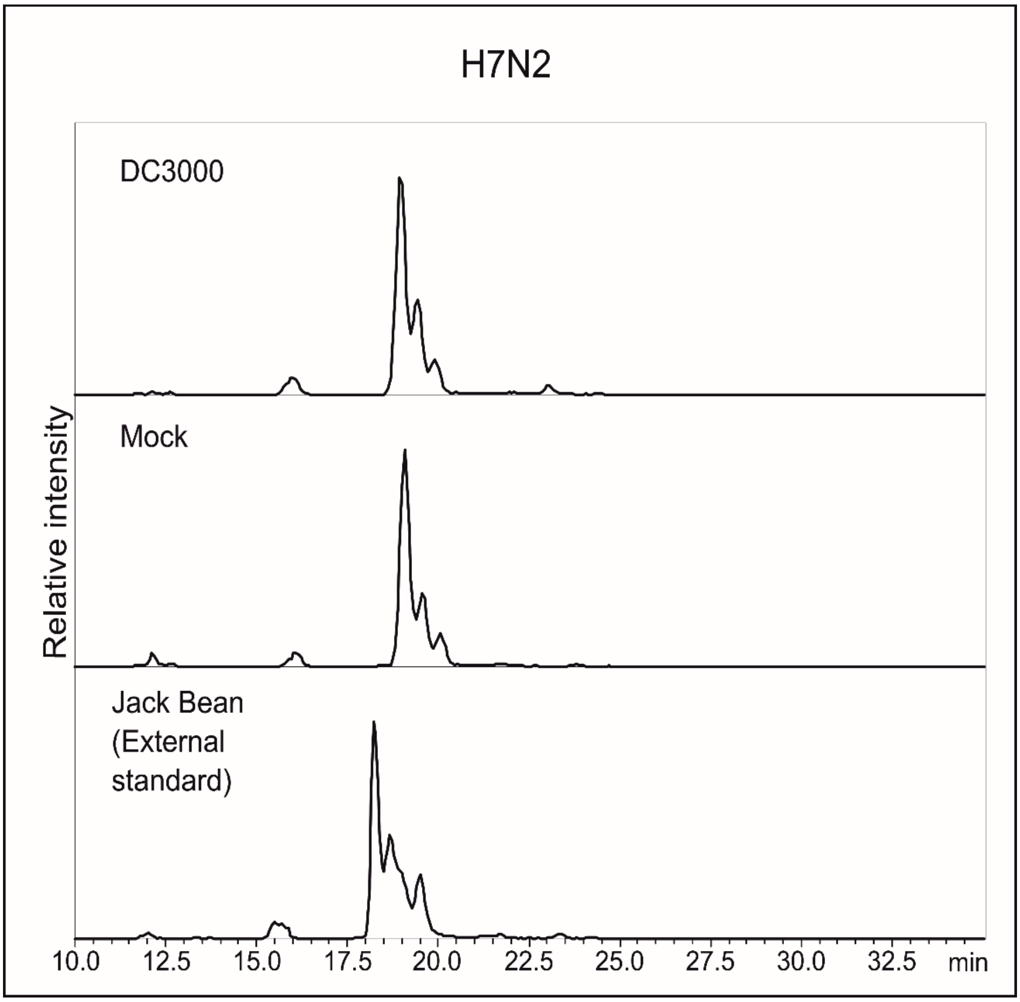

Figure S7. PGC-MS analysis of H7N2 glycans from Col-0 wild-type seedlings either mock-treated or DC3000-infected. Samples were harvested 3 dpi and N-glycans purified. N-glycans from jack bean were used as external standard. Due to the low resolution, the individual peaks could not be assigned to N-glycan isomers.

Table S1. List of relative amounts of individual N-glycans in infected and mock treated Col-0 plants at 1, 3 and 5 dpi. Numbers indicate percentage of individual glycoforms in respect to total N-glycans ± standard deviation from four independent biological replicates. The quantification of individual N-glycans is based on the HPLC-FLD measurement.

	**Mock 1 dpi**	**Mock 3 dpi**	**Mock 5 dpi**	**DC3000 1 dpi**	**DC3000 3 dpi**	**DC3000 5 dpi**
**H11N2**					0.82 ± 0.24	0.53 ± 0.22
**H10N2**	1.00 ± 0.16	0.56 ± 0.34	0.66 ± 0.06	1.16 ± 0.17	2.22 ± 0.39	1.81 ± 0.57
**H9N2**	6.83 ± 0.61	6.15 ± 0.70	6.35 ± 1.03	6.94 ± 0.52	5.87 ± 0.41	5.25 ± 0.79
**H9N2***					3.20 ± 0.28	2.19 ± 0.50
**H8N2**	7.27 ± 0.40	7.11 ± 0.80	6.76 ± 0.53	7.31 ± 0.38	6.92 ± 0.58	6.76 ± 0.78
**H8N2***					5.23 ± 0.13	4.34 ± 0.46
**H7N2**	6.17 ± 0.28	7.06 ± 1.23	6.47 ± 0.27	5.66 ± 0.14	4.78 ± 0.28	5.16 ± 0.33
**H6N2**	5.96 ± 0.14	6.97 ± 0.92	6.40 ± 0.19	5.83 ± 0.14	4.41 ± 0.29	4.55 ± 0.60
**H5N2**	8.50 ± 0.95	9.80 ± 0.97	9.03 ± 3.05	9.00 ± 0.99	7.23 ± 0.48	7.76 ± 1.19
**H4N2**				0.56 ± 0.57	3.03 ± 1.03	2.54 ± 0.54
**H3N4XF**	22.1 ± 1.46	20.3 ± 0.74	19.5 ± 1.55	18.7 ± 0.49	8.93 ± 0.35	7.92 ± 0.39
**H3N3XF**	9.91 ± 0.49	9.13 ± 0.50	10.7 ± 2.49	9.96 ± 0.85	7.98 ± 1.08	7.70 ± 0.38
**H3N2XF**	29.9 ± 1.20	29.9 ± 3.71	31.5 ± 1.73	32.1 ± 1.56	34.8 ± 2.50	40.0 ± 2.82
**H3N2X**	2.35 ± 0.64	3.06 ± 0.61	2.73 ± 0.65	2.87 ± 0.90	4.62 ± 0.60	3.48 ± 1.17
